# Alterations of the expression of T-cell-related costimulatory CD28 and downregulatory CD152 (CTLA-4) molecules in patients with B-cell chronic lymphocytic leukaemia

**DOI:** 10.1038/sj.bjc.6601833

**Published:** 2004-04-27

**Authors:** I Frydecka, A Kosmaczewska, D Bocko, L Ciszak, D Wolowiec, K Kuliczkowski, I Kochanowska

**Affiliations:** 1 Institute of Immunology and Experimental Therapy, Polish Academy of Sciences, R Weigla 12, 53-114 Wroclaw, Poland; 2 Department of Haematology, Medical University, Pasteura 4, 50-367 Wroclaw, Poland

**Keywords:** B-CLL, CD152 (CTLA-4), CD28, stimulation

## Abstract

In the present study, we have examined the kinetics and magnitude of expression of the CD28 and CD152 molecules on unstimulated and anti-CD3+rIL-2-stimulated peripheral blood CD4+ and CD8+ T cells in patients with chronic lymphocytic leukaemia (B-CLL) and controls. The mean percentages of both CD3+/CD4+/CD28+ and CD3+/CD8+/CD28+ cells were significantly lower in B-CLL than in controls before culture, decreased rapidly, reaching their lowest levels between 24 and 48 h, and returned to basal levels after 72 h of culture. In controls, the lowest proportions of CD3+/CD4+/CD28+ and CD3+/CD8+/CD28+ cells were found after 24 h and returned to prestimulation levels after 48 h of stimulation. We observed significantly higher proportions of unstimulated CD3+/CD4+/CD152+ and CD3+/CD8+/CD152+ cells in B-CLL patients than in controls. The highest percentages of CD3+/CD4+/CD152+ and CD3+/CD8+/CD152+ cells were observed in controls after 72 h, and in B-CLL patients after 24 h, and remained statistically higher after 48, 72 and 96 h of stimulation. CD152 molecule expression returned to prestimulation levels after 96 h of culture in controls, and after 120 h in B-CLL patients. The abnormal kinetics and levels of CD28 and CD152 expression on T cells in B-CLL may lead to a state of hyporesponsiveness or anergy and could be one of the mechanisms of immune deficiency in this disease.

B-cell chronic lymphocytic leukaemia (B-CLL) is the most frequent form of adult leukaemia in the Western world, where it accounts for about 25% of all leukaemias. In addition to the accumulation and clonal expansion of malignant B cells, several abnormalities have been demonstrated within the non-malignant T-cell population. The nature and the level of T-cell immune deficiency in B-CLL are the object of intense investigations. Recently, there has been a growing appreciation of the importance of the costimulatory and inhibitory regulation pathways in normal and disease-related cellular immune function.

The effective activation of naive T lymphocytes requires the generation of a specialised contact area between the T cell and the antigen-presenting cells (APCs) known as the immunologic synapse (Dustin and Cooper, 2000). This area of interaction is generated by the aggregation of different specialised receptors and signalling molecules, called supramolecular activation clusters. The differentiation and expansion of a T cell depends upon two signals: an antigen-specific interaction between the T–cell receptor (TCR) and the specific peptide embedded in major histocompatibility complex (MHC) molecules displayed on the surface of the APC, and an antigen-nonspecific interaction between a costimulatory receptor and its ligand. CD28 is the primary T-cell costimulatory molecule, which is expressed constitutively on almost all CD4+ T cells and on about 50% of CD8+ T cells. Upon interaction with the ligands B.7-1 (CD80) and/or B.7-2 (CD86), CD28 transduces a signal that enhances T-cell proliferation and cytokine secretion and sustains T-cell response (reviewed in Bocko *et al*, 2002). In the absence of an appropriate costimulation, TCR occupancy alone can lead to T-cell unresponsiveness or clonal anergy, in which T cells are unable to proliferate or secrete cytokines in response to a secondary stimulation (Schwartz, 1996). Consistent with this, CD28-negative transgenic mice exhibit profound defects in mitogenic responses (Shahinian *et al*, 1993), and germinal centres are not formed in response to immunisation (Ferguson *et al*, 1996).

Conversely, the CD28 homologue CD152 (CTLA-4: cytotoxic T-lymphocyte-associated antigen-4), which is transiently expressed on activated T cells, reaching its highest level after 48–72 h of stimulation (Walunas *et al*, 1994; Kosmaczewska *et al*, 2002), plays an inhibitory role in regulating T-cell activation. The expression of CD152 within T-cell population is restricted to the subset of T cells that also express CD28 on the cell surface (Lindsten *et al*, 1993). CD152 binds the same ligands like CD28, but has an affinity 10–50-fold higher than the CD28 receptor for B.7-1 (CD80) and B.7-2 (CD86) molecules (Linsley *et al*, 1994). CD152 mRNA and protein synthesis is induced by the ligation of CD28 with its ligands (Lindsten *et al*, 1993). CD152 engagement inhibits the induction of the IL-2 receptor alpha chain (CD25), CD69 molecule expression, CD3/CD28-induced IL-2 mRNA accumulation, and secretion of IL-2 (Krummel and Allison, 1996; Blair *et al*, 1998). CD152 ligation also augments the production of transforming growth factor beta (TGF-beta) (Chen *et al*, 1998). Mice lacking CD152 as a result of targeted gene disruption develop a fatal spontaneous lymphoproliferative disease with massive lymphocyte infiltrates in many organs (Waterhouse *et al*, 1995; Tivol *et al*, 1997).

The outcome of an immune response involves a balance between CD28–mediated T-cell activation and CD152-mediated inhibition. Little is known about CD28 and CD152 expression on peripheral blood (PB) T cells in patients with B-CLL. To the best of our knowledge, only three papers have been published on the expression of these molecules in B-CLL patients so far (Rossi *et al*, 1996; Van den Hove *et al*, 1998; Scrivener *et al*, 2001). Our study was designed to evaluate the kinetics and expression of CD28 and CD152 on unstimulated and anti–CD3+rIL-2-stimulated CD4+ and CD8+ T lymphocytes from B-CLL patients.

## MATERIALS AND METHODS

### Patients

Samples from 33 untreated patients aged 33–80 years (mean: 64. 6±10.5 years) fulfilling the morphologic and immunophenotypic criteria for the diagnosis of B-CLL were studied. Patients were graded according to Rai's staging system as at stages III (17 cases) and IV (16 cases). The control samples consisted of PB from 25 age- and sex-matched healthy individuals.

The study was approved by the local research ethics committee.

### Isolation of peripheral blood mononuclear cells and culture conditions

Peripheral blood mononuclear cells (PBMCs) were separated from freshly drawn heparinised peripheral venous blood of the B-CLL patients and healthy donors by buoyant density gradient centrifugation on Lymphoflot (Biotest AG, Germany) and washed three times in 0.9% saline. Peripheral blood mononuclear cells were suspended at 1 × 10^6^ PBMCs ml^−1^ in RPMI 1640 medium (Gibko, Paisley, UK) supplemented with 10% foetal calf serum (Flow Labs, UK), L-glutamine and 50 *μ*g ml^−1^ gentamycin (Gibko), and cultured with 5 ng ml^−1^ of anti-CD3 monoclonal antibodies (MoAbs) (Ortho, Neckargemund, Germany) and 500 U ml^−1^ of rIL-2 (Eurocetus, Amsterdam, The Netherlands). In our model, rIL-2 served as a second signal to induce optimal immune response. Control cultures without stimulants were included in each experiment. The cultures were incubated at 37°C in a humidified atmosphere containing 5% CO_2_ for 24, 48, 72, 96 and 120 h.

### Flow cytometric analysis

All experiments on the fresh and cultured cells were carried out by triple labelling with anti-CD152 (CTLA-4)/RPE (PharMingen, Becton Dickinson Company, San Diego, CA, USA), anti-CD3/PerCP (Becton Dickinson, San Jose, CA, USA), anti-CD4/FITC (Becton Dickinson, San Jose, CA, USA), anti-CD8/FITC (Becton Dickinson, San Jose, CA, USA), anti-CD28/FITC (Serotec, UK), anti–CD4/RPE (Becton Dickinson, San Jose, CA, USA) and anti-CD8/RPE (Becton Dickinson, San Jose, CA, USA) MoAbs.

Briefly, the cells were incubated for 30 min at 4°C with the antibodies described above, and excess, unbound antibodies were removed by two washes with PBS containing 0.5% Tween-20. Following these washes, the cells were fixed with PBS (without Ca^2+^ and Mg^2+^) and analysed by flow cytometry using a FACScalibur flow cytometer (Becton Dickinson, Mountain View, CA, USA). Negative controls were always done by omitting the MoAb as well as by incubating cells with mouse Ig of the same isotype as the MoAbs conjugated with fluoresceine or phycoerythrin.

The results were expressed as the proportions of CD3+/CD4+ and CD3+/CD8+ cells coexpressing CD28 or CD152 antigen. At least 10 000 events per sample were analysed in triple staining analysis. The CellQuest program was used for statistical analysis of the acquired data.

### Statistical analysis

Statistical analysis was performed using Mann–Whitney *U*-test and ANOVA test for repeated measurements. Differences were considered as statistically significant when the *P*-value was ⩽0.05. Summary statistics are given as the mean ±s.d.

## RESULTS

### The level and kinetics pattern of CD28 expression on CD3+/CD4+ and CD3+/CD8+ T cells in B-CLL patients and controls

The mean percentage of CD3+/CD4+ cells coexpressing CD28 was significantly lower in the B-CLL patients than in the controls before stimulation (*P*=0.0002) and after 24 h (*P*=0.0001), 48 h (*P*=0.0001), 72 h (*P*=0.0001) and 96 h (*P*=0.0001) of *ex vivo* stimulation (Table 1

**Table 1:** The mean percentage of PB CD3+/CD4+/CD28+ and CD3+/CD8+/CD28+ cells before and after 24, 48, 72 and 96 h of anti-CD3+rIL-2 stimulation in patients with B-CLL and healthy controls

	**Time (h)**					
	**0**	**24**	**48**	**72**	**96**	***P***
*CD3+/CD4+/CD28+*						
						
B-CLL patients (*n*=33)	41.5±17.1%	20.1±9.2%	22.1±9.9%	35.7±13.5%	36.2±12.6%	I : III=0.001
	(I)	(III)	(V)	(VII)	(IX)	I : V=0.005
						III : V=NS
						V : VII=0.001
						
Healthy controls (*n*=25)	78.9±7.6%	57.0±8.5%	74.7±6.5%	82.2±6.0%	82.1±6.4%	II : IV=0.002
	(II)	(IV)	(VI)	(VIII)	(X)	IV : VI=0.004
						II : VI=NS
						VI : VIII=NS
*P*	I : II=0.0002	III : IV=0.0001	V : VI=0.0001	VII : VIII=0.0001	IX : X=0.0001	
						
*CD3+/CD8+/CD28+*						
						
B-CLL patients (*n*=33)	36.3±17.6%	20.3±12.2%	25.1±18.4%	41.0±15.7%	40.2±11.1%	I : III=0.04
	(I)	(III)	(V)	(VII)	(IX)	I : V=0.05
						III : V=NS
						V : VII=0.02
						
Healthy controls (*n*=25)	59.3±9.8%	42.6±11.2%	52.5±8.3%	54.7±8.3%	55.0±7.6%	II : IV=0.04
	(II)	(IV)	(VI)	(VIII)	(X)	IV : VI=0.05
						II : VI=NS
						VI : VIII=NS
*P*	I : II=0.005	III : IV=0.001	V : VI=0.001	VII : VIII=0.01	IX : X=0.01	

). The mean percentage of CD3+/CD8+/CD28+ cells was also significantly lower in B-CLL patients than in controls before stimulation (*P*=0.005), and after 24 h (*P*=0.001), 48 h (*P*=0.001), 72 h (*P*=0.01) and 96 h (*P*=0.01) of stimulation (Table 1). The lowest proportions of CD3+/CD4+/CD28+ and CD3+/CD8+/CD28+ cells in B-CLL patients were observed between 24 and 48 h of stimulation, and returned to basal levels after 72 h of culture. In healthy subjects, the lowest proportions of CD3+/CD4+/CD28+ and CD3+/CD8+/CD28+ cells were observed after 24 h and returned to prestimulation levels after 48 h (Figure 1

**Figure 1:**
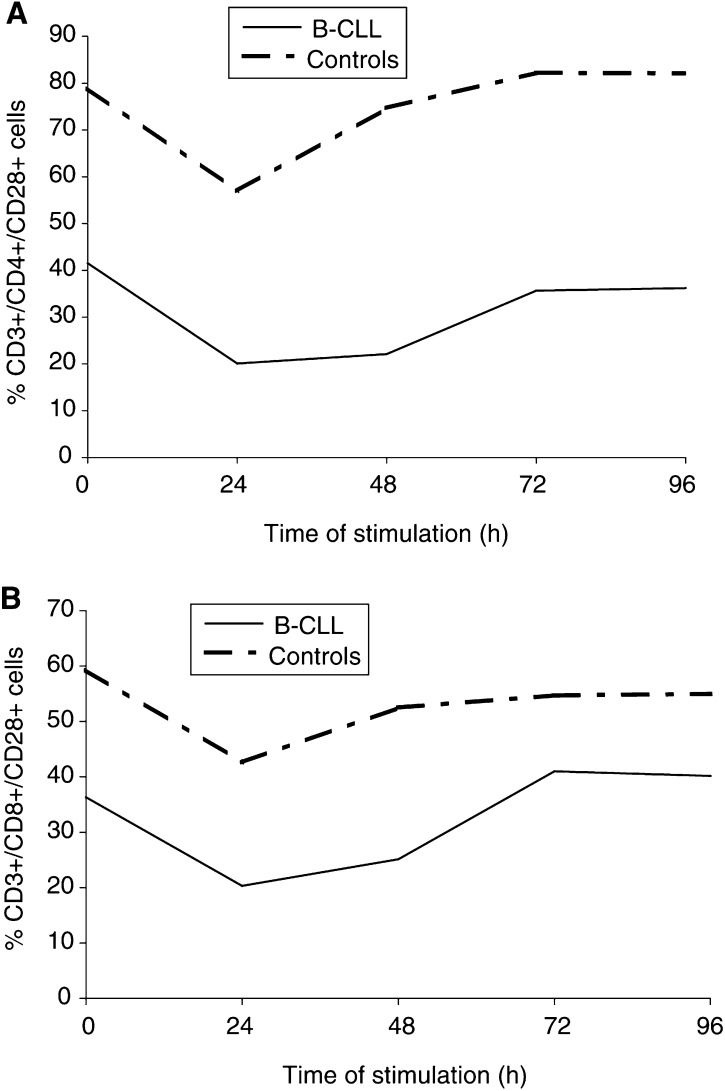
The mean percentage of PB CD3+/CD4+/CD28+ cells (**A**) and CD3+/CD8+/CD28+ cells (**B**) before and after *ex vivo* 24, 48, 72 and 96 h of anti–CD3+rIL-2 stimulation in patients with B-CLL and normal subjects.

).

The mean fluorescence intensity (MFI) of the CD28+ cells, as a measure of the antigen density on the cell surface, was significantly lower on CD4+ T cells in B-CLL patients compared with controls after 48 h (*P*=0.01) and 72 h (*P*=0.03) (Table 2

**Table 2:** MFI values of CD28 on PB CD3+/CD4+ cells and CD3+/CD8+ cells, expressed in arbitrary units (AU), before and after 24, 48, 72 and 96 h of anti-CD3+rIL-2 stimulation in patients with B-CLL and healthy controls

	**MFI of CD28**				
	**0 h**	**24 h**	**48 h**	**72 h**	**96 h**
*CD3+/CD4+*					
B-CLL patients (*n*=33)	41.0±29.4	39.4±14.7	47.4±30.0	36.8±29.8	49.4±33.8
Healthy controls (*n*=25)	38.2±11.8	47.5±4.6	65.0±17.8	65.3±11.8	55.2±13.5
*P*	NS	NS	0.01	0.03	NS
					
*CD3+/CD8+*					
B-CLL patients (*n*=33)	44.7±21.1	45.3±18.5	52.0±25.0	42.1±31.7	48.7±32.6
Healthy controls (*n*=25)	40.8±31.3	53.3±28.4	75.5±41.1	69.9±33.1	59.8±37.6
*P*	NS	NS	NS	0.03	NS

), while significantly lower MFI values of CD28 on CD3+/CD8+ cells compared with controls were found only after 72 h of stimulation (*P*=0.03) (Table 2).

### The level and kinetics pattern of CD152 expression on CD3+/CD4+ and CD3+/CD8+ T cells in B-CLL patients and controls

The frequency of CD3+/CD4+/CD152+ cells was significantly higher in B-CLL patients than controls on freshly drawn PB cells (*P*=0.001) and after 24 h (*P*=0.0001), 48 h (*P*=0.0001), 72 h (*P*=0.0005), 96 h (*P*=0.001) and 120 h (*P*=0.001) of stimulation (Table 3

**Table 3:** The mean percentage of PB CD3+/CD4+/CD152+ cells and CD3+/CD8+/CD152+ cells before and after 24, 48, 72, 96 and 120 h of anti-CD3+rIL-2 stimulation in patients with B-CLL and healthy controls

	**Time (h)**						
	**0**	**24**	**48**	**72**	**96**	**120**	***P***
*CD3+/CD4+/CD152+*							
							
B-CLL patients (*n*=33)	11.8±7.2%	19.8±11.0%	19.0±9.3%	18.2±8.2%	14.7±4.0%	9.9±4.2%	I : III=0.002
	(I)	(III)	(V)	(VII)	(IX)	(XI)	I : V=0.003
							III : V=NS
							V : VII=NS
							VII : IX=0.03
							IX : XI=0.01
							
Healthy controls (*n*=25)	3.0±0.8%	4.7±1.0%	7.2±2.0%	10.4±2.9%	3.2±0.5%	2.2±0.4%	II : IV=0.002
	(II)	(IV)	(VI)	(VIII)	(X)	(XII)	IV : VI=0.001
							II : VI=0.0003
							VI : VIII=0.01
							VIII : X=0.0001
							X : XII=NS
							
*P*	I : II=0.001	III : IV=0.0001	V : VI=0.0001	VII : VIII=0.0005	IX : X=0.001	XI : XII=0.001	
CD3+/CD8+/CD152+							
							
B-CLL patients (*n*=33)	12.5±7.8%	20.0±15.0%	20.7±10.5%	19.7±9.4%	17.5±8.0%	11.5±5.1%	I : III=0.02
	(I)	(III)	(V)	(VII)	(IX)	(XI)	I : V=0.002
							III : V=NS
							V : VII=NS
							VII : IX=NS
							IX : XI=0.0001
							
							
Healthy controls (*n*=25)	2.9±1.3%	6.1±2.0%	10.0±3.2%	12.0±3.9%	3.2±1.1%	2.0±0.7%	II : IV=0.008
	(II)	(IV)	(VI)	(VIII)	(X)	(XII)	IV : VI=0.004
							II : VI=0.0001
							VI : VIII=0.05
							VIII : X=0.0001
							X : XII=NS
*P*	I : II=0.0001	III : IV=0.0005	V : VI=0.0003	VII : VIII=0.02	IX : X=0.0001	XI : XII=0.0001	

).

The mean percentage of CD3+/CD8+/CD152+ cells was also markedly elevated in B-CLL compared with controls before stimulation (*P*=0.0001) and after 24 h (*P*=0.0005), 48 h (*P*=0.0003), 72 h (*P*=0.02), 96 h (*P*=0.0001) and 120 h (*P*=0.0001) of stimulation (Table 3).

**Figure 2:**
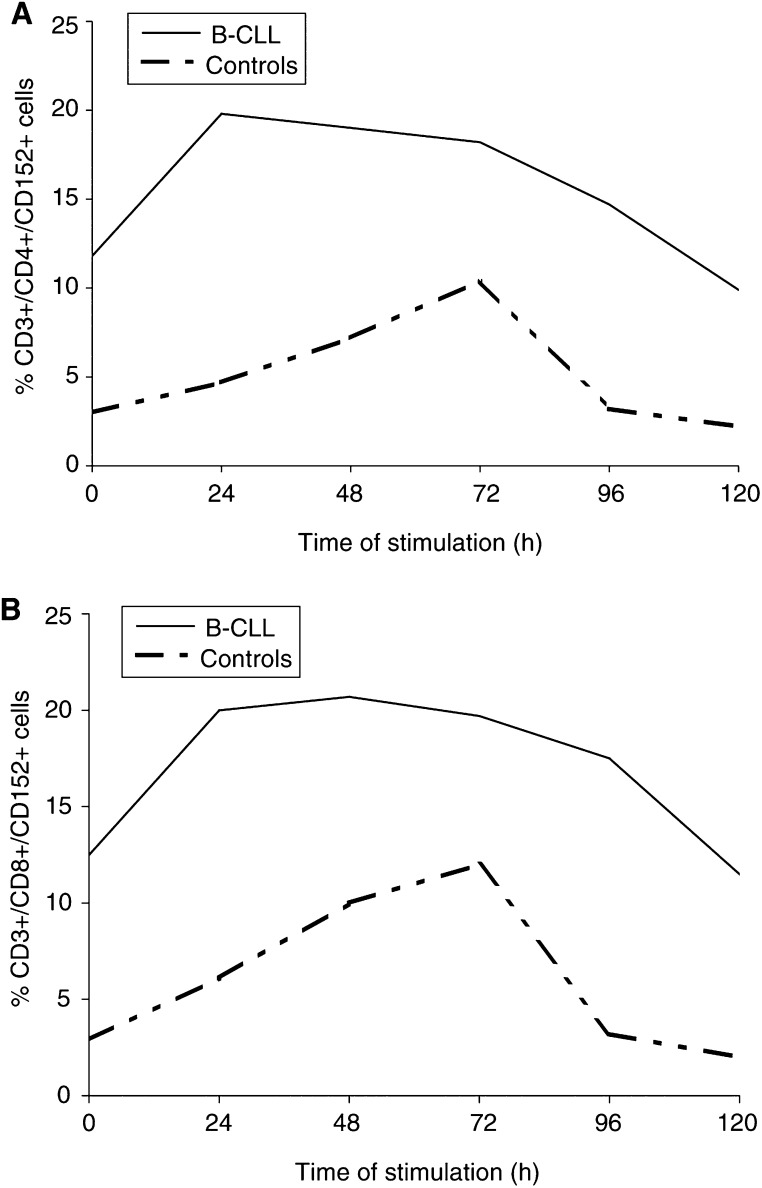
The mean percentage of PB CD3+/CD4+/CD152+ cells (**A**) and CD3+/CD8+/CD152+ cells (**B**) before and after *ex vivo* 24, 48, 72, 96 and 120 h of anti–CD3+rIL-2 stimulation in patients with B-CLL and normal subjects.

In healthy subjects, the proportions of both CD3+/CD4+/CD152+ and CD3+/CD8+/CD152+ cells increased gradually, peaked after 72 h after stimulation, and returned to basal levels after 96 h of stimulation. In contrast, in B-CLL patients, the highest proportion of triple-positive cells was observed after 24 h of stimulation, slowly decreased on subsequent days of stimulation, and returned to prestimulation levels after 120 h of culture (Figure 2

).

In addition to the increased frequency of CD3+/CD4+/CD152+ cells, we also observed significantly higher MFI values of CD152 on CD3+/CD4+ in B-CLL patients compared with controls on unstimulated (*P*=0.001) as well as on stimulated cells after 24, 48, and 72 h of culture (*P*=0.04, 0.01 and 0.03, respectively) (Table 4

**Table 4:** MFI values of CD152 on PB CD3+/CD4+ cells and CD3+/CD8+ cells, expressed in arbitrary units (AU), before and after 24, 48, 72, 96 and 120 h of anti-CD3+rIL-2 stimulation in patients with B-CLL and healthy controls

	**MFI of CD152**					
	**0 h**	**24 h**	**48 h**	**72 h**	**96 h**	**120 h**
*CD3+/CD4+*						
B-CLL patients (*n*=33)	252±124	232±73	243±75	260±92	206±49	219±74
Healthy controls (*n*=25)	167±39	172±30	154±31	189±73	164±48	172±64
*P*	0.001	0.04	0.01	0.03	NS	NS
						
*CD3+/CD8+*						
B-CLL patients (*n*=33)	302±168	304±159	317±129	251±77	205±79	227±58
Healthy controls (*n*=25)	165±36	223±46	198±54	198±34	202±55	180±64
*P*	0.01	0.05	0.01	0.05	NS	NS

). Similar results were obtained in the CD3+/CD8+ subpopulation on unstimulated (*P*=0.01) and stimulated cells 24 h (*P*=0.05), 48 h (*P*=0.01) and 72 h (*P*=0.05) after stimulation (Table 4).

### Comparison between CD28 as well as CD152 expressions on CD4+ and CD8+ T cells before and after *ex vivo* stimulation in healthy donors and B-CLL patients

In the control group, a significantly higher proportion of CD3+/CD4+/CD28+ than CD3+/CD8+/CD28+ cells was found before and after 24, 48, 72 and 96 h of stimulation (*P*=0.0005, 0.0005, 0.0008, 0.0001 and 0.0001, respectively). Mean fluorescence intensity values did not differ between the subsets of lymphocytes studied.

In the case of CD152 molecule expression, markedly higher proportions of CD3+/CD8+/CD152+ than CD3+/CD4+/CD152+ cells after 24 and 48 h of stimulation (*P*=0.05 and 0.04, respectively) as well as higher MFI values tested at the same time points (*P*=0.01 and 0.04, respectively) were found.

In contrast, in B-CLL patients, there were similar frequencies of CD3+/CD4+/CD28+ and CD3+/CD8+/CD28+ cells (Table 1) as well as CD3+/CD4+/CD152+ and CD3+/CD8+/CD152+ cells (Table 3). Similarly, there were no marked differences between the MFI values of both the CD28+ cells and CD152+ cells within the subsets of T cells studied in these patients.

## DISCUSSION

There are only a few reports regarding CD28 expression on unstimulated and stimulated T lymphocytes in B-CLL. However, no data concerning the kinetics pattern of the studied molecules on the subsets of T cells in B-CLL patients and controls have been reported so far. Rossi *et al* (1996) found a significant decrease in CD28 molecules on T cells in 33 patients with B-CLL. Similar results were obtained by Van den Hove *et al* (1998) in 12 B-CLL patients. In a recent study, Scrivener *et al* (2001) reported a decreased proportion of CD2+/CD28+ cells, which did not change upon 4 h of stimulation with OKT3 MoAb in 27 patients with B-CLL. The same authors also found a complete lack of CD152 expression on freshly drawn PB T-cells of half of the patients. The 48 h *ex vivo* stimulation with OKT3 MoAb or PHA increased the mean proportion of CD2+/CD152+ from 1.9±2.7 to 6.8±5.1%. In the present study, we demonstrated for the first time abnormal levels and a different kinetics pattern of costimulatory CD28 and inhibitory CD152 molecules expression on *ex vivo*-stimulated CD3+/CD4+ and CD3+/CD8+ PB T cells in B-CLL patients compared with healthy controls.

Since an analysis of CD28-positive cells within the CD8 population would be complicated by the fact that the CD8 subset is comprised of both CD3+ T lymphocytes and CD3− NK cells (Perussia *et al*, 1983), we performed our studies on CD4+ and CD8+ T cells using a triple immunostaining method (a T-cell marker, T-cell-subset markers, and CD28 or CD152). In B-CLL patients, the proportions of CD28+ cells within the CD3+/CD4+ and CD3+/CD8+ populations before and during stimulation were significantly lower at each time point tested, and more pronounced in CD4+ T cells compared with controls. After stimulation in the control subjects, we found, similar to other reports (Linsley *et al*, 1993), a transient decrease of CD28 expression on both subsets of T cells after 24 h, which returned to the prestimulation level after 48 h. In the B-CLL patients, the lowest proportions of both subsets of CD28-positive T cells were observed between 24 and 48 h and returned to basal levels after 72 h of *ex vivo* stimulation. In addition to the decreased frequency of CD3+/CD4+/CD28+ and CD3+/CD8+/CD28+ cells in B-CLL patients, the MFI of the CD28+ cells, as a measure of the antigen density on the cell surface, was also lower in patients at 48 and 72 h on CD4+ T cells and 72 h on CD8+ T cells after stimulation than in the controls.

The mechanisms underlying the abnormalities in CD28 expression in B-CLL patients are not fully understood. The CD28 molecule is lost by normal lymphocytes after repeated stimulation with IL-2 in long-term culture (Labalette *et al*, 1999). Based on the fact that T lymphocytes from B-CLL patients have a phenotype of activated cells, that is, HLA-DR+, CD38+, CD45RO+, CD11c+, CD69+, CD71+, CD40L+ (Dianzani *et al*, 1994; Van den Hove *et al*, 1998; Scrivener *et al*, 2001), it can be suggested that the loss of CD28 molecule on T lymphocytes before culture is related to a prolonged *in vivo* activation of these cells. Our finding of a markedly increased expression of the inducible suppressory CD152 molecule on freshly drawn CD4+ and CD8+ T cells in B-CLL patients strengthens the suggestion that T cells in B-CLL are in a partial state of activation. The loss of CD28 expression on B-CLL T cells may be also related to the influence of the elevated TNF-alpha serum levels produced by neoplastic B lymphocytes and T cells in patients with B-CLL (Adami *et al*, 1994; Bojarska-Junak *et al*, 2002; Gallego *et al*, 2003). It has been shown that exposure of T cells to TNF-alpha leads to the direct repression of the transcriptional activity of the CD28 gene promoter (Bryl *et al*, 2001).

The mechanisms leading to the prolonged downregulation of CD28 expression after *ex vivo* stimulation in B-CLL patients is difficult to explain. The changes in CD28 expression kinetics due to differential dynamics of proliferation of CD28-negative T cells seem unlikely, since these cells display a poor proliferative capacity, which cannot be overcome by the addition of exogenous IL-2 (Azuma *et al*, 1993). The fact that the reversion of CD28 took place rapidly (between 24 and 48 h of stimulation) favours the interpretation that in B-CLL patients the prolonged downregulation of the surface CD28 molecule may result from enhanced ligation-stimulated CD28 receptor endocytosis, and/or disturbed recycling to the cell surface or increased proteolytic intracellular degradation.

The expression and kinetics pattern of the CD152 molecule on PB CD4+ and CD8+ T cells after stimulation also differed markedly from that observed in normal subjects. The frequencies of CD3+/CD4+/CD152+ and CD3+/CD8+/CD152+ cells were significantly higher at each time point tested compared with normal subjects. After stimulation in normal subjects, the highest proportions of T cells coexpressing CD152 molecule were found after 72 h of culture, which is similar to the findings of others (Walunas *et al*, 1996) and our own previous report (Kosmaczewska *et al*, 2002). In patients with B-CLL, the highest proportions of CD4+ T cells and CD8+ T cells coexpressing CD152 were observed after 24 h and returned to basal levels after 120 h, but after 96 h in controls. The results of our present study showing the abnormal kinetics and expression of CD28 on T cells in B-CLL may provide a possible explanation for the observed alterations in CD152 expression after *ex vivo* stimulation. It has been established that the physiological downregulation of CD28 expression at both the mRNA and protein levels during the first 24 h of stimulation rapidly and strongly enhances transcription of the *CTLA-4* gene (Lindsten *et al*, 1993; Linsley *et al*, 1993). We suggest that the significantly lower CD28 antigen expression on both subsets of unstimulated T cells and its more profound and long-lasting downregulation after stimulation compared with normal controls as observed in our study may deliver a stronger and prolonged stimulus for CD152 induction and expression on the CD3+/CD4+ and CD3+/CD8+ T-cell subpopulations in B-CLL. Since CD152 inhibits T-cell responses, increased expression of CD152 molecule on both subsets of T cells may result in an impairment of T-cell function in patients with B-CLL (Bartik *et al*, 1998; Lee *et al*, 1998; Metzler *et al*, 1999; Carreno *et al*, 2000; Frydecka *et al*, 2003; Wolowiec *et al*, 2003). This hypothesis was confirmed by our previously reported results, which showed strong negative correlations between the proportion of PB CD3+/CD152+ cells and proliferative activity, IL-2 and IFN-γ production in patients with Hodgkin's disease and healthy subjects (Kosmaczewska *et al*, 2002).

In summary, the dysregulated expression and kinetics of the costimulatory CD28 and downregulatory CD152 molecules on PB T cells of patients with B-CLL may likely have a considerable impact on the biology of T-cell responses and could be one of the mechanisms of immune deficiency in this disease (Bartik *et al*, 1998). Therapeutic manipulations of the B-7 : CD28 : CD152 costimulatory and inhibitory pathways may provide a potential avenue for increasing T-cell responses in B-CLL patients.
